# A Pilot Randomized Controlled Trial of Extracorporeal Vaginal Peflex Weights for Enhancing Pelvic Floor Function and Relieving Stress Urinary Incontinence

**DOI:** 10.3390/ijerph22111703

**Published:** 2025-11-11

**Authors:** Avital Bar Chen, Tal Fligelman, Leonid Kalichman

**Affiliations:** 1 Department of Physical Therapy, Recanati School for Community Health Professions, Faculty of Health Sciences, Ben Gurion University of the Negev, P.O. Box 653, Beer-Sheva 8410501, Israel; 2 Meuhedet Health Services, Central District, Tel Aviv 6266106, Israel

**Keywords:** stress urinary incontinence, pelvic floor muscle training, Peflex weights, randomized controlled trial, muscle endurance and power

## Abstract

Background: Stress urinary incontinence (SUI) is the involuntary loss of urine during increased abdominal pressure, affecting 46% of adult women, particularly those over 40. Pelvic floor muscle (PFM) training is the first-line treatment supported by numerous high-quality studies. However, the effectiveness of biofeedback devices, such as vaginal weight cones, remains controversial. Peflex weights are a new type of vaginal extracorporeal weights developed for PFM training. Aims: To evaluate the effectiveness of PFM training with Peflex weights in reducing SUI symptoms, improving muscle power and endurance, and comparing its efficacy to standard PFM training without weights. Methods: A pilot randomized controlled trial was conducted involving 35 women aged 18 to 50 diagnosed with SUI. Participants were randomly assigned to either the Peflex group (PFM training with Peflex weights) or the control group (PFM training without weights). Both groups engaged in six weeks of home-based training. The primary outcome was assessed using the self-reported International Consultation on Incontinence Questionnaire-Short Form (ICIQ-UI-SF). Secondary outcomes included evaluations based on the PERFECT scheme, perineometer measurements, and levator hiatus diameters obtained via ultrasound. Results: In the intragroup analysis, the Peflex group significantly improved all outcome measurements. Compared to the control group, the Peflex group demonstrated significantly higher improvement in muscle power and repetition of muscle contraction (*p* = 0.015 and *p* = 0.007, respectively), as well as in the proportional change in levator hiatus contraction (*p* = 0.022). There was no significant difference in the improvement in ICIQ-UI-SF and perineometer measurements between the groups (*p* > 0.05). Additionally, there was a trend of higher satisfaction with the treatment in the Peflex group (*p* = 0.054). Conclusions: Peflex weights effectively reduce SUI symptoms and improve muscle power and endurance, with high user satisfaction. However, there was no significant difference in the main outcome measure (ICIQ-UI-SF) between the Peflex and control groups. Further research is needed to identify which patients benefit most from this treatment.

## 1. Introduction

Stress urinary incontinence (SUI) is a prevalent disorder defined by the involuntary loss of urine during activities that increase intra-abdominal pressure, such as coughing, laughing, or sneezing [1,2]. It is estimated to affect around 46% of adult women, with prevalence rising notably with age [3]. The condition primarily arises from two mechanisms: urethral hypermobility, resulting from weakened support structures of the bladder and urethra [4], and intrinsic sphincter deficiency, which occurs when diminished urethral mucosal or muscular tone impairs the urethra’s ability to close effectively [5]. Several risk factors contribute to SUI development. Pregnancy increases bladder pressure due to both weight gain and altered posture associated with a shifted center of gravity [6]. Vaginal delivery further heightens the risk—approximately doubling it compared with cesarean section [7]—by overstretching the pelvic floor muscles (PFM), compromising bladder neck support, and potentially damaging pudendal nerve integrity [3]. Elevated body mass index (BMI) is another important factor; excess abdominal fat raises intra-abdominal pressure, which subsequently increases urethral mobility and stress on pelvic support structures [8].

Pelvic floor muscle (PFM) training remains the gold standard and first-line conservative management for SUI, supported by level 1A evidence [9]. Research consistently shows that PFM exercise enhances muscle tone, optimizes neuromuscular recruitment, prevents PFM descent under pressure, and ultimately reduces leakage episodes. The goals of this training are to improve contraction coordination, muscle strength and endurance, and the passive stiffness of pelvic floor tissues [10]. Exercise frequency plays a critical role; training at least three times per week has been shown to yield significantly greater strength gains compared to less frequent sessions [11]. PFM programs typically consist of repetitive voluntary contractions that progressively improve muscle tone and perineal support [12].

The inclusion of biofeedback—whether via perineometer or electromyographic (EMG) systems—has been demonstrated to further improve training efficacy by providing real-time feedback on muscle activation and contraction quality [13,14,15]. Another tool for pelvic floor rehabilitation is the use of vaginal cones, which serve as both a biofeedback and resistance device. The principle behind them is straightforward: when the cone begins to slip from the vagina, the user instinctively contracts the pelvic muscles to prevent expulsion [16]. Although studies have shown some benefits, their superiority over standard PFM training remains debated [17,18,19].

**Figure 1:**
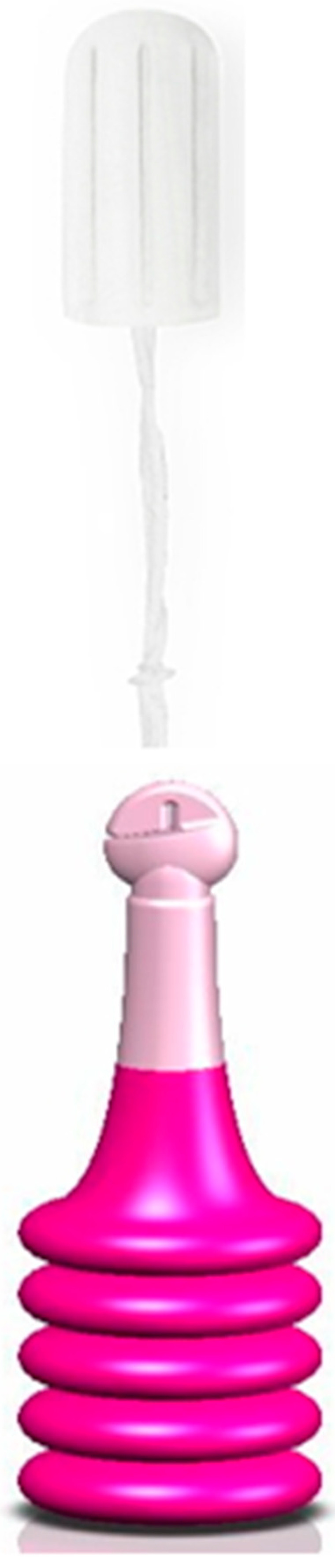
Peflex.

A recent development in this field is the Peflex device, a system employing extracorporeal vaginal weights (Figure 1). Similar in concept to traditional vaginal cones, it introduces adjustable external loads attached to a tampon-like string, allowing individualized resistance and enhanced sensorimotor feedback. Developed by pelvic floor physiotherapist Ira Kushnir, Peflex has gained increasing popularity and is now widely available for patient use and professional pelvic floor training programs.

This study evaluates the efficiency of PFM training with Peflex weights in reducing SUI symptoms. We hypothesized that training with vaginal extracorporeal Peflex weights would enhance symptoms of SUI, boost muscle power and endurance, and serve as a safe and enjoyable tool for practice.

## 2. Methods

### 2.1. Design

This was a pilot prospective single-blinded randomized controlled trial conducted on women with SUI symptoms who attended the Meuhedet Women’s Health Medical Center, Tel Aviv, Pinkas clinic for 24 months.

### 2.2. Subjects

Subjects with complaints of SUI were referred to the physical therapist by a board-certified urogynecologist, who made the diagnosis of SUI.

Inclusion criteria: Age: 18–50, diagnosed with SUI after at least one birth (either vaginal or cesarean section), symptoms prevalent for over six weeks.

Exclusion criteria included menopause, pregnancy, vaginal dryness or infection, recurrent urinary tract infections, hypertonic PFM, dyspareunia, grade III prolapse, use of medications that may affect the symptoms of SUI, either directly or indirectly by promoting or inhibiting diuresis, neurological impairments involving the central nervous system, connective tissue disorders, and inability to correctly contract the PFM by the end of the initial physical therapy session.

### 2.3. Ethical Considerations

The physical therapist conducting the study screened potential participants based on the inclusion and exclusion criteria. Eligible individuals were invited to participate and provided a detailed explanation of the study’s aims and procedures. Subjects who agreed to participate were asked to sign an informed consent form. The study received approval from the Meuhedet Ethical (Helsinki) committee (approval number 02-25-05-22).

### 2.4. Procedures and Data Collection

During the first meeting, participants received verbal explanations about pelvic floor anatomy, muscle localization, and function using anatomic models and illustrations. The participants underwent a complete evaluation by the physical therapist who conducted this study.

The *PFM contractility evaluation* was performed through digital palpation. The participants were placed in the supine position, with their knees flexed (60–90° degrees as comfortable) and their feet on the surface. The examiner used disposable gloves and lubricating gel, and the index and middle fingers were introduced 2–3 cm into the vagina; the participant was asked to perform a maximum contraction of the muscles, lifting inward and squeezing around the fingers. Muscle contractility was graded according to the PERFECT scheme (Version 2001) [20], according to the definitions by Laycock and Jerwood: P = Power; E = endurance; R = repetitions of item F = fast contractions; ECT = every contraction timed. The PERFECT scheme underwent a validation process. The bi-digital examination of inter-rater reliability was considered moderate for power: κw = 0.57, endurance: ICC = 0.53 and fast contractions: ICC = 0.65, and low for repetitions: ICC = 0.27 [20].

To assess *power*, participants were instructed to perform voluntary strong contractions. The power was assessed using the Modified Oxford Grading Scale (zero to five points): 1—minor muscle flicker, 2—weak muscle activity, 3—moderate muscle contraction, 4—good muscle contraction, and 5—strong muscle contraction.

To assess *endurance*, participants were instructed to perform a sustained voluntary contraction with the same degree of strength as assessed by the Oxford scale, with a maximum time of 10 s. To assess repetitions, participants repeated the contraction performed in endurance, with a 4 s rest between each repetition, with a maximum of ten repetitions. Fast contractions assessed how many contractions the participant was able to perform vigorously and quickly with the same initial power strength, with a maximum of ten contractions being considered. They had a one-minute rest between the evaluations of each item. ECT reminds examiners to time all previously assessed items and does not require scoring [20,21].

*The PFM strength* was also measured by using a perineometer (Guangzhou Ibeier Technology) [22,23]. It is a conical insert probe, 28 mm in diameter and 108 mm in length, covered in a thin, medical-grade silicon rubber sheath. The vaginal insert probe is connected to a microprocessor that allows the transmission of pressure readings in centimeters of water (cmH_2_O) when the insert is compressed by external pressure. Participants were asked to lie supine with bent knees (60–90° as comfortable). The vaginal pressure probe was placed inside the vagina, where 0.5 to 1 cm of the insert was visible outside the body. The best result of 3 evaluations was recorded [24]. The perineometer was validated with a high-reliability ICC = 0.88 [25].

*Urogynecological symptoms assessment*: Participants were asked to complete the International Consultation on Incontinence Questionnaire Urinary Incontinence—Short Form (ICIQ-UI-SF). It is used to evaluate the frequency, severity, and impact on quality of life regarding urinary incontinence in men and women. It is scored on a scale from 0–21 [26]. The ICIQ-UI-SF is considered a valid, reliable, and robust tool [27].

**Figure 2:**
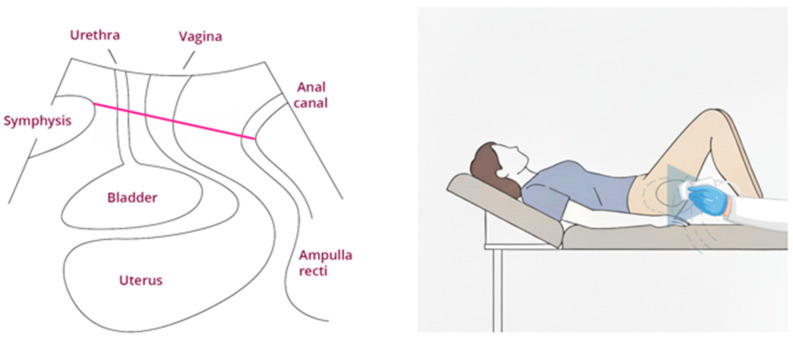
Mid-sagittal two-dimensional (2D) pelvic floor measurement was taken to determine levator hiatus diameters.

*Assessment of the levator hiatus* proportional change in contraction: Participants were supine, their knees flexed (60–90° as comfortable), with an empty bladder. The transducer of ultrasound was covered with a condom and gel, put on the labia, and a mid-sagittal two-dimensional (2D) pelvic floor measurement was taken to determine levator hiatus diameters (Figure 2). The mid-sagittal plane identifies the plane of minimal hiatal dimensions, evident as the minimal distance between the hyperechogenic posterior aspect of the symphysis pubis and the hyperechogenic anterior border of the puborectalis muscle. The measurement was taken at rest and in contraction. A 15 s video clip of the perineum at rest and in contraction was recorded. The video was saved on the ultrasound device. The physical therapist who conducted this study evaluated the levator hiatus proportional change in contraction: Proportional change in contraction = (Rest-contract)/Rest × 100 [28,29,30]. The proportional change in 2D levator hiatal AP diameter was validated with a high-reliability ICC = 0.81 [29].

### 2.5. Allocation

At the end of the baseline evaluation, participants were randomly allocated to intervention and control groups. Sixty sealed envelopes were prepared in advance with cards with the names of the study groups inside, 30 for the control group and 30 for the intervention group. All sealed envelopes were thoroughly mixed. The subject randomly picked up the consequent envelope and gave it to the physical therapist. The participants were blinded to the allocation. The participants were aware of the type of treatment but unaware of the study group, whether control or intervention.

### 2.6. Interventions

All participants were instructed on how to perform the PFM training program. The Peflex group was instructed to exercise with Peflex weights, whereas the control group exercised without the use of instruments. The Peflex device has five weights. Each weight is 50 g, and a total of 5 weights is 250 g, which can hang on a hidden spring. The Peflex device is not inserted into the vagina but is hung off the string of an inserted tampon. The amount of the Peflex weights was determined before starting the exercise program. The patient inserted a tampon and attached a 50 g Peflex weight. The participant was then asked to contract the pelvic floor while standing. If the contraction produced even minimal upward movement of the spring—a visible displacement indicating activation of the pelvic floor muscles—the weight was considered appropriate for training. Additional weights were gradually added until the participant could no longer lift the device. The final starting weight was defined as the heaviest load the participant could lift with minimal effort. If the participant demonstrated signs of excessive effort, compensatory movements (such as hip or abdominal activation), inability to lift, or early fatigue, the weight was reduced. The goal was to determine the maximum weight the participant could lift while completing at least 70% of the prescribed training protocol. During the initial supervised session, all participants in the intervention group were able to complete the full protocol. Both groups received an identical explanatory page with a list of exercises to perform; they also received a practice diary to monitor the amount of training the subject did. The intervention group performed precisely the same training but with the Peflex weights. All participants received bi-weekly reminders via telephone call or WhatsApp message to exercise. For the Peflex group, after three weeks, the weight of the Peflex was increased according to the patient’s capabilities, as reported during the phone call. Both groups continued the training program for six weeks and returned for the final meeting to do a second complete examination. The physical therapist who conducted the study was blinded to the first evaluation; a second blinded repeated US measurements evaluation was performed, and the analysis was made on the second evaluation.

### 2.7. Intervention Protocol

The training program was conducted for six weeks, four times a week, for 10 min. The exercise was carried out in two static positions and three dynamic positions. The static positions are sitting and standing. The patient was asked to do three sets of ten fast contractions at maximal strength with 10 s of relaxation between sets and three sets of ten-second prolonged contractions at maximal strength with 10 s of relaxation between sets. The dynamic positions are standing and moving the pelvis in a circular movement with prolonged contraction at maximal strength for 10 s; in addition, the patient was asked to do ten squats with pelvic floor contractions at maximal strength each time (relax the pelvic floor between each squat). Ten rises on the fingertips with pelvic floor contraction at maximal strength each time (relax the pelvic floor between each rise); for training the pelvic floor while increasing abdominal pressure, the patient was asked to do ten fast contractions at maximal strength while coughing in a standing position (1 contraction per cough).

### 2.8. Statistical Analysis

Data analysis was performed using IBM SPSS Statistics 29 software. Descriptive statistics were employed to summarize the demographic characteristics and baseline measurements of the participants. The Shapiro–Wilk test was conducted to assess the normality of the data, revealing a non-normal distribution.

Baseline differences between groups were evaluated using the Mann–Whitney U test for continuous variables and the Chi-square test (or Fisher’s exact test, as appropriate) for categorical variables. Intra-group comparisons (pre- and post-intervention) were conducted using the Wilcoxon signed-rank test, while inter-group comparisons (differences in outcomes between groups) were analyzed using the Mann–Whitney U test. Statistical significance was set at *p* < 0.05.

## 3. Results

Thirty-five women were recruited at the Meuhedet Pincas Center in Tel Aviv, Israel. The study was carried out between July 2022 and July 2024. The participants were randomly divided into 20 in the Peflex group and 15 in the control group of PFM training. Three (8.5%) participants from the Peflex group dropped out of the study; they did not attend the second meeting for various reasons, such as losing the Peflex, not having time, or not wanting to do the exercise. After excluding these cases, 32 participants completed the trial: 17 participants in the Peflex group and 15 in the control group. Four participants were excluded from the final statistical evaluation because they did not adhere to the training protocol: three from the Peflex group and one from the control group. Two participants in the Peflex group did not have the time to follow the training protocol four times a week; instead, they practiced only once a week. Additionally, one subject reported difficulty using the tampon, which is necessary for use with the Peflex. One subject from the control group did not have the time to train four times a week; she trained less than twice a week. The exclusion process resulted in 14 participants remaining in each group.

During the study, the participants did not report any side effects or complications from using the Peflex weight or the training protocol. The demographic characteristics of the participants are described in Table 1.

### 3.1. Power Analysis

A post hoc power analysis was conducted for the primary outcome variable, the ICIQ-UI-SF, to evaluate the statistical power of this pilot study. The analysis was based on observed mean and standard deviation values for the Peflex and Control groups. Key parameters for the analysis included a significance level of 0.05, a desired power of 0.80, and a two-tailed test.

The Peflex group showed a mean reduction in ICIQ-UI-SF scores from 10.07 (SD = 2.64) pre-treatment to 5.50 (SD = 3.44) post-treatment, while the Control group’s scores reduced from 12.29 (SD = 3.10) pre-treatment to 7.36 (SD = 3.27) post-treatment (Table 2). Using these observed differences, an effect size of approximately 1.5 was calculated for both groups, reflecting a substantial improvement in symptoms. Based on the calculated effect size and sample size of 14 participants per group, the achieved power for detecting differences in ICIQ-UI-SF scores was 0.83. This indicates sufficient power to identify significant improvements within and between groups for this outcome measure.

For the secondary outcomes, the observed improvements in perineometer pressure and levator hiatus proportional change were also analyzed:

*Perineometer Pressure*: The Peflex group achieved a moderate improvement compared to the control group. The calculated power for this secondary outcome was approximately 0.79, reflecting sufficient ability to detect a moderate effect.

*Levator Hiatus Proportional Change*: Significant differences were observed in the Peflex group compared to the control group. The achieved power for this measure was approximately 0.85, confirming adequate sensitivity to detect these differences.

Although the study demonstrated sufficient power for some outcomes, the small sample size limited its ability to detect smaller effects, particularly in the control group for certain measures. These findings highlight the potential for Peflex training to produce clinically relevant improvements and emphasize the need for larger trials to confirm these results and explore their broader applicability.

### 3.2. Primary Outcome Measures: Intra-Group Comparison

At baseline, no significant differences were observed between the groups in any outcome measures (Table 2). After six weeks of training, both groups showed significant within-group improvements in total ICIQ-UI-SF scores: from 10.07 ± 2.64 to 5.50 ± 3.44 in the Peflex group (Z = −3.071, *p* = 0.002) and from 12.29 ± 3.10 to 7.36 ± 3.27 in the Control group (Z = −2.350, *p* = 0.002).

Analysis of the individual questionnaire items revealed that the amount of incontinence significantly decreased only in the Peflex group (2.71 ± 0.99 → 1.57 ± 0.85; *p* = 0.004), while no significant change occurred in the Control group (*p* = 0.102). The frequency of incontinence declined significantly in both groups (*p* = 0.005 and *p* = 0.025, respectively). The daily-life interference score also improved significantly in both groups (*p* = 0.005 and *p* = 0.003, respectively).

All comparisons were analyzed using the Wilcoxon signed-rank test.

### 3.3. Secondary Outcome Measures: Intra-Group Comparison

At baseline, there were no significant differences between groups in any secondary outcomes (Table 3). After six weeks of training, the Peflex group showed significant within-group improvements in all parameters—perineometer pressure, power, endurance, repetitions, fast contractions, and ultrasound-measured levator hiatus contraction (all *p* < 0.05).

In the Control group, significant improvements were observed in perineometer pressure, power, endurance, repetitions, and fast contractions (*p* < 0.05 for all), but not in the levator hiatus proportional change (*p* = 0.331).

Perineometer pressure increased from 12.79 ± 12.56 to 17.77 ± 14.49 cmH_2_O in the Peflex group (*p* = 0.001) and from 11.07 ± 7.19 to 13.43 ± 7.36 cmH_2_O in the Control group (*p* = 0.019). Strength (Oxford scale), endurance, repetitions, and fast contractions all improved significantly in both groups (*p* < 0.05). The levator hiatus contraction amplitude increased significantly only in the Peflex group (7.64 ± 5.18 → 10.71 ± 5.61 mm; *p* = 0.048).

The comparison between the groups was based on the mean percentage of improvement, calculated as the difference between post- and pre-intervention measurements after six weeks of training.

All statistical analyses were performed using the Wilcoxon signed-rank test.

### 3.4. Primary and Secondary Outcome Measures: Inter-Group Comparison

The comparison of primary and secondary outcomes between groups is presented in Table 4. Data are shown for the Peflex group (N = 14) and the Control group (N = 14). The comparison between the groups was based on the mean percentage of improvement (the measurement obtained after six weeks of training minus the measurement obtained before the intervention).

Significant inter-group differences were found in power, repetitions, and levator hiatus proportional change in contraction, all favoring the Peflex group. No significant differences were observed for the other parameters, including total ICIQ-UI-SF score, daily life interference, frequency and amount of incontinence, perineometer pressure, endurance, or fast contractions.

The mean percentage change in power was 9.09 ± 5.04 in the Peflex group versus 3.89 ± 5.87 in the Control group (U = 50.00, *p* = 0.015). Repetitions improved by 39.61 ± 21.27% compared with 17.20 ± 15.96% (U = 40.00, *p* = 0.007). The levator hiatus proportional contraction increased by 3.06 ± 5.52% in the Peflex group and decreased by −1.17 ± 3.54% in the Control group (U = 48.00, *p* = 0.022).

For all other outcomes, including total ICIQ-UI-SF score, perineometer pressure, endurance, and fast contractions, the inter-group differences were not statistically significant (all *p* > 0.05). Treatment satisfaction scores were comparable between groups (8.64 ± 1.50 vs. 7.50 ± 1.51; U = 57.00, *p* = 0.054).

All inter-group comparisons were performed using the Mann–Whitney U test.

## 4. Discussion

This pilot prospective randomized controlled trial studies the effectiveness of vaginal extracorporeal weights (Peflex) on SUI symptoms severity, PFM strength, and treatment satisfaction.

### 4.1. Intragroup Comparison

In the Peflex group, the total ICIQ-UI-SF significantly improved compared to the baseline. The frequency of SUI, amount of incontinence, and quality of life also significantly improved. PFM strength, measured by a perineometer, increased significantly after the Peflex training protocol. The PERFECT scheme showed significant improvement in muscle power, endurance, and fast contraction. US levator hiatus measurements showed a significant improvement in the proportional contraction change. These findings align with previous studies on pelvic floor training as a first-line treatment for SUI [9]. The PFM training protocol included repeated concentric contractions and sustained contractions in vertical body positions and whole-body functional exercises like squats, rise on tiptoes, and rotatory motion of the pelvis. The Peflex training was efficient for treating SUI, similar to previous studies on Vaginal Cone Therapy [31]. The improvement in US levator hiatus measurements correlates with earlier studies on pelvic floor contraction [28].

Significant improvement was also found in most measurements in the control group. However, after six weeks of training, there was no significant reduction in the amount of incontinence displayed in the ICIQ-UI-SF questionnaire and no improvement in the US levator hiatus measurement of the proportional change of contraction. These findings differ from other studies, possibly due to the shorter duration of our study (six weeks) compared to others (six months) [17,32].

### 4.2. Comparison Between the Groups

We aimed to determine if PFM training with Peflex weights would have the same or more beneficial effects than training without Peflex weights in women with SUI. The Peflex group significantly improved in power and repetition of muscle contraction, and the levator hiatus proportional change in contraction (*p* < 0.05). There was a trend of more treatment satisfaction in the Peflex group (*p* = 0.054). However, there was no significant difference in the improvement in SUI symptoms based on the ICIQ-UI-SF questionnaire, similar to other studies comparing vaginal cones and EMG biofeedback training or pelvic floor muscle training [19,33,34]. Some studies found significant differences in outcome measures between cone and PFM training groups [32,35].

The muscle power and repetition increased significantly in the Peflex group after only six weeks of training compared to the control group. The benefits of Peflex may include the following:

- The Peflex weights, as opposed to vaginal weights, are not inserted into the vagina but hung on a tampon string; the Peflex weights are pulled to the ground by gravity, unlike the vaginal cone that lies in a transverse position and is therefore retained in the vagina [36].
- The Peflex has a spring that allows for a slight vibrating movement that stimulates the PFM’s proprioception, which stimulates the muscle to contract; it is known that neural deficits may lead to SUI [37]. There is evidence that whole-body vibration training increases the strength of the PFM in women with SUI [38] and that proprioceptive input influences muscle activity timing, posture, coordination, and balance [39].
- The Peflex allows for visual biofeedback by practicing in front of a mirror and sensory biofeedback by pulling the Peflex down. The Peflex enables adjustments in the levels of training difficulty, such as altering the size of a tampon (the smaller the tampon, the more complex the training), adding more weights, and modifying the body’s training position (sitting, standing, squatting, etc.). The training can be more functional when carried out on a full bladder. It is recommended that training be conducted during the activity in which the patient reports SUI dysfunction, such as while singing.
- All the above induce sensorimotor adaptation, promoting pelvic floor reaction and enhancing motor learning. Motor learning encompasses repeated practice with successful outcomes, visual feedback that allows one to observe the weight being lifted upward, and practicing pelvic floor contractions with varying weights and positions [40].
- Relaxation is essential in muscle training. Muscle relaxation enables the muscles to recover between contractions, facilitating optimal strength development [41]. Reaching this state of relaxation is recommended by holding and lifting the Peflex weights.

Overall, this study shows that Peflex weights are safe and do not have side effects. Training with Peflex weights significantly decreases SUI symptoms (*p* = 0.002) and significantly increases muscle power (*p* = 0.015) and repetitions (*p* = 0.007) compared to training the PFM without Peflex weights. However, there was no significant difference between the groups in the reduction of SUI symptoms as measured by the ICIQ-UI-SF questionnaire (*p* = 0.660). This can be explained by the variant causes of SUI, as this training program dealt only with muscle impairment. The PFM are striated muscles containing slow-twitch and fast-twitch fibers, essential for maintaining continence [41,42]. Other factors contributing to SUI include anatomical defects, nerve damage, and reduced reflex contraction of the pelvic muscles [37].

To sum up, while Peflex weights offer a promising non-surgical option for treating SUI, treatment success depends on selecting the right patient. Women who feel discomfort using a tampon might struggle with adherence to the Peflex program. Certain medical conditions might also make using Peflex inappropriate (such as pelvic inflammatory disease, vaginal infections, or severe pelvic organ prolapse). Nevertheless, women who are physically able and motivated to perform regular PFM exercises will benefit from the enhanced muscle training that Peflex provides. Further research with a larger sample size is needed to enhance the study’s power and better understand the mechanical and physiological effects of Peflex. Additionally, it is essential to study the benefits of Peflex weights on menopausal women, women with pelvic prolapse, and men with SUI after prostatectomy.

### 4.3. Possible Limitations

This study has several limitations that should be considered when interpreting the findings. First, the physical therapist conducting the outcome assessments was not blinded to group assignments, which may have introduced observer bias. Second, while significant differences were observed in some secondary outcomes favoring the Peflex group, no significant differences were found in the primary outcome measure (ICIQ-UI-SF). This may be attributed to the small sample size, which limits the statistical power and generalizability of the results.

Additionally, outcome measurements were conducted in a supine position, which may not fully capture SUI symptoms that typically occur in upright or dynamic activities. This methodological aspect might have underestimated the true functional improvements achieved through the interventions.

As this was a pilot study, the findings provide valuable preliminary data but warrant further investigation through larger-scale trials with longer follow-up periods to enhance the robustness and generalizability of the results.

## 5. Conclusions

This pilot study demonstrates the potential of Peflex weights as a promising, non-surgical tool for pelvic floor muscle training in women with SUI. Training with Peflex weights significantly improved pelvic floor muscle power, endurance, and the repetition of muscle contractions compared to standard pelvic floor muscle training without weights. The Peflex group also demonstrated higher treatment satisfaction and a significant improvement in the proportional contraction of the levator hiatus, reflecting enhanced muscle responsiveness.

While both groups experienced reductions in SUI symptoms, no significant difference was observed between the groups in the primary outcome measure, the ICIQ-UI-SF. This suggests that the short duration of the study or the variability in SUI etiology might have limited the ability to detect broader benefits.

The Peflex weights proved to be safe, with no reported adverse effects, making them a feasible option for pelvic floor rehabilitation. However, adherence to the Peflex training program is crucial, and future studies should explore strategies to improve user compliance and determine optimal patient selection criteria.

Further research with a larger sample size and a longer follow-up period is necessary to confirm the observed benefits and clarify the mechanical and physiological effects of Peflex weights.

## Figures and Tables

**Table 1:** The demographic characteristics of the participants.

Demographic Data	Peflex Group (N = 14)	Control Group (N = 14)	*p*-Value
Age (years)			0.645 ^^
Mean ± SD	40.93 ± 5.60	39.50 ± 7.39	
BMI (kg/m^2^)			0.713 ^^
Mean ± SD	23.11 ± 2.86	23.91 ± 3.26	
Number of deliveries			0.266 ^^
Mean ± SD	2.36 ± 1.39	2.00 ± 0.43	
Median (Q1, Q3)	2.00 (1.00, 6.00)	1.50 (1.00, 7.00)	
Newborn weight (grams)			0.679 ^^
Mean ± SD	3272.86 ± 366.76	3286.86 ± 741.62	
Number of pregnancies			0.538 ^^
Mean ± SD	3.14 ± 2.24	2.57 ± 1.95	
Median (Q1, Q3)	2.00 (1.00, 7.00)	2.00 (1.00, 8.00)	
Instrumental birth			~1.000 ^
Yes	2 (14%)	3 (21%)	
No	12 (86%)	11 (79%)	
Cesarean delivery			0.481 ^
Yes	2 (14%)	0 (0%)	
No	8 (86%)	14 (100%)	
Obstetric rupture			~1.000 ^
Yes	8 (57%)	9 (64%)	
No	6 (43%)	5 (36%)	
Episiotomy			~1.000 ^
Yes	6 (42%)	6 (42%)	
No	8 (58%)	8 (58%)	
Weekly sports activity (hours)			0.303 ^^
Mean ± SD	2.00 ± 1.24	1.43 ± 1.45	

^ Fisher’s exact test, ^^ Mann–Whitney, SD—standard deviation.

**Table 2:** The primary outcome measurement; intra-group comparison.

Outcome Measures	Peflex Group			Control Group		
	Pre-Treatment Mean ± SD	Post-Treatment Mean ± SD	*p*-Value	Pre-Treatment Mean ± SD	Post-Treatment Mean ± SD	*p*-Value
Total ICIQ-UI-SF score	10.07 ± 2.64	5.50 ± 3.44	0.002 **	12.29 ± 3.10	7.36 ± 3.27	0.002 **
Amount of incontinence	2.71 ± 0.99	1.57 ± 0.85	0.004 **	2.57± 1.22	2.00 ± 0.00	0.102
Frequency of incontinence	1.86 ± 0.86	0.86 ±0.53	0.005 **	2.21 ± 1.05	1.29 ± 0.83	0.025 **
Daily Life Interference (NRS)	5.64± 1.65	3.07 ± 2.53	0.005 **	7.50 ± 2.21	4.07 ± 2.64	0.003 **

** Wilcoxon, statistically significant differences are marked in bold. SD—standard deviation.

**Table 3:** The secondary outcome measures of the study: intra-group comparison.

Outcomes Measures	Peflex Group			Control Group		
	Pre-Treatment Mean ± SD	Post-Treatment Mean ± SD	*p*-Value	Pre-Treatment Mean ± SD	Post-Treatment Mean ± SD	*p*-Value
Perineometer pressure (cmH_2_O)	12.79 ± 12.56	17.77 ± 14.49	0.001 **	11.07 ± 7.19	13.43 ± 7.36	0.019 **
Power (1–5)	2.5 ± 1.09	3.57 ± 0.85	0.027 **	2.64 ± 0.84	3.07 ± 0.83	0.034 **
Endurance (seconds)	8.07 ± 2.59	9.86 ± 0.53	0.001 **	5.79 ± 2.83	8.79 ± 1.67	0.003 **
Repetitions (N)	3.79 ± 1.85	8.14 ± 1.83	0.004 **	4.79 ± 2.42	6.64 ± 2.44	0.005 **
Fast contractions (N)	5.71 ± 2.40	8.93 ± 1.86	0.004 **	6.36 ± 2.44	8.21 ± 2.04	0.029 **
US levator hiatus. Proportional change in contraction (mm)	7.64 ± 5.18	10.71 ± 5.61	0.048 **	8.26 ± 7.34	7.08 ± 5.90	0.331

** Wilcoxon, statistically significant differences are marked in bold. SD—standard deviation. US—ultrasound.

**Table 4:** The outcome measures of the study, comparison between groups.

Outcomes Measures	Peflex Group Mean ± SD	Control Group Mean ± SD	*p*-Value
Relative Delta Total ICIQ-UI-SF score (%)	41.55 ± 31.43	−44.80± 35.91	0.660
Delta Frequency incontinence (%)	−9.09 ± 7.13	−8.44 ± 11.53	0.883
Delta Amount of incontinence (%)	−10.38 ± 9.33	−5.19 ± 11.11	0.096
Delta Daily Life Interference (%)	−23.37 ± 20.39	−31.16 ± 26.13	0.471
Delta Perineometer (%)	37.01 ± 2993	21.42 ± 28.66	0.179
Delta Power (%)	9.09 ± 5.04	3.89 ± 5.87	0.015 **
Delta Endurance (%)	16.23 ± 23.43	27.27 ± 19.20	0.121
Delta Repetitions (%)	39.61 ± 21.27	17.20 ± 15.96	0.007 **
Delta Fast contractions (%)	29.22 ± 24.23	16.88 ± 26.40	0.286
Delta Levator hiatus Proportional change in contraction (%)	3.06 ± 5.52	−1.17 ± 3.54	0.022 **
Satisfaction with treatment (NRS)	8.64 ± 1.50	7.50 ± 1.51	0.054

** Mann–Whitney, statistically significant differences are marked in bold. SD—standard deviation.

## Data Availability

The raw data supporting the conclusions of this article will be made available by the authors on request.

## References

[1] Vaughan C.P., Markland A.D. (2020). Urinary incontinence in women. Ann. Intern. Med..

[2] Haylen B.T., de Ridder D., Freeman R.M., Swift S.E., Berghmans B., Lee J., Monga A., Petri E., Rizk D.E., Sand P.K. (2010). An International Urogynecological Association (IUGA)/International Continence Society (ICS) joint report on the terminology for female pelvic floor dysfunction. Int. Urogynecol. J..

[3] Abufaraj M., Xu T., Cao C., Siyam A., Isleem U., Massad A., Soria F., Shariat S.F., Sutcliffe S., Yang L. (2021). Prevalence and trends in urinary incontinence among women in the United States, 2005–2018. Am. J. Obstet. Gynecol..

[4] Elia D., Gambiacciani M., Ayoubi J.-M., Berreni N., Bohbot J.M., Descamps P., Druckmann R., Geoffrion H., Haab F., Heiss N. (2020). Female urine incontinence: Vaginal erbium laser (VEL) effectiveness and safety. Horm. Mol. Biol. Clin. Investig..

[5] Wu J.M. (2021). Stress incontinence in women. N. Engl. J. Med..

[6] Barakat R., Pelaez M., Montejo R., Luaces M., Zakynthinaki M. (2011). Exercise during pregnancy improves maternal health perception: A randomized controlled trial. Am. J. Obstet. Gynecol..

[7] Tähtinen R.M., Cartwright R., Tsui J.F., Aaltonen R.L., Aoki Y., Cárdenas J.L., El Dib R., Joronen K.M., Al Juaid S., Kalantan S. (2016). Long-term Impact of Mode of Delivery on Stress Urinary Incontinence and Urgency Urinary Incontinence: A Systematic Review and Meta-analysis. Eur. Urol..

[8] Subak L.L., Richter H.E., Hunskaar S. (2009). Obesity and urinary incontinence: Epidemiology and clinical research update. J. Urol..

[9] Abrams P., Andersson K., Apostolidis A., Birder L., Bliss D., Brubaker L., Cardozo L., Castro-Diaz D., O’COnnell P., Cottenden A. (2018). 6th international consultation on incontinence. Recommendations of the international scientific committee: Evaluation and treatment of urinary incontinence, pelvic organ prolapse and faecal incontinence. Neurourol. Urodyn..

[10] Hay-Smith E.J.C., Dumoulin C. (2006). Pelvic floor muscle training versus no treatment, or inactive control treatments, for urinary incontinence in women. Cochrane Database Syst. Rev..

[11] García-Sánchez E., Ávila-Gandía V., López-Román J., Martínez-Rodríguez A., Rubio-Arias J.Á. (2019). What Pelvic Floor Muscle Training Load is Optimal in Minimizing Urine Loss in Women with Stress Urinary Incontinence? A Systematic Review and Meta-Analysis. Int. J. Environ. Res. Public Health.

[12] Felicíssimo M.F., Carneiro M.M., Saleme C.S., Pinto R.Z., da Fonseca A.M.R.M., da Silva-Filho A.L. (2010). Intensive supervised versus unsupervised pelvic floor muscle training for the treatment of stress urinary incontinence: A randomized comparative trial. Int. Urogynecol. J..

[13] Bertotto A., Schvartzman R., Uchôa S., Wender M.C.O. (2017). Effect of electromyographic biofeedback as an add-on to pelvic floor muscle exercises on neuromuscular outcomes and quality of life in postmenopausal women with stress urinary incontinence: A randomized controlled trial. Neurourol. Urodyn..

[14] Herderschee R., Hay-Smith E.C.J., Herbison G.P., Roovers J.P., Heineman M.J. (2013). Feedback or biofeedback to augment pelvic floor muscle training for urinary incontinence in women: Shortened version of a Cochrane systematic review. Neurourol. Urodyn..

[15] Nunes E.F.C., Sampaio L.M.M., Biasotto-Gonzalez D.A., Nagano R.C.D.R., Lucareli P.R.G., Politti F. (2019). Biofeedback for pelvic floor muscle training in women with stress urinary incontinence: A systematic review with meta-analysis. Physiotherapy.

[16] Kato K., Kondo A. (1997). Clinical value of vaginal cones for the management of female stress incontinence. Int. Urogynecol. J. Pelvic Floor. Dysfunct..

[17] Castro R.A., Arruda R.M., Zanetti M.R.D., Santos P.D., Sartori M.G.F., Girão M.J.B.C. (2008). Single-blind, randomized, controlled trial of pelvic floor muscle training, electrical stimulation, vaginal cones, and no active treatment in the management of stress urinary incontinence. Clinics.

[18] Golmakani N., Khadem N., Arabipoor A., Kerigh B.F., Esmaily H. (2014). Behavioral Intervention Program versus Vaginal Cones on Stress Urinary Incontinence and Related Quality of Life: A Randomized Clinical Trial. Oman Med. J..

[19] Sahin N., Yesil H., Gorcan B. (2022). The effect of pelvic floor exercises performed with EMG biofeedback or a vaginal cone on incontinence severity, pelvic floor muscle strength, and quality of life in women with stress urinary incontinence: A randomized, 6-month follow-up study. Int. Urogynecol. J..

[20] da Silva J.B., de Godoi Fernandes J.G., Caracciolo B.R., Zanello S.C., de Oliveira Sato T., Driusso P. (2021). Reliability of the PERFECT scheme assessed by unidigital and bidigital vaginal palpation. Int. Urogynecol. J..

[21] Ahlund S., Nordgren B., Wilander E.-L., Wiklund I., Fridén C. (2013). Is home-based pelvic floor muscle training effective in treatment of urinary incontinence after birth in primiparous women? A randomized controlled trial. Acta Obstet. Gynecol. Scand..

[22] Ouchi M., Kitta T., Takahashi Y., Chiba H., Higuchi M., Togo M., Shinohara N. (2020). Reliability of manometry for assessing pelvic floor muscle function in healthy men. Neurourol. Urodyn..

[23] Bele A., Qureshi M. (2021). Impact of Electrotherapy or Muscle Training on Quality of Life in Urinary Incontinence of Male Geriatric Population-A Protocol. J. Clin. Diagn..

[24] Tosun O.C., Mutlu E.K., Ergenoglu A., Yeniel A., Tosun G., Malkoc M., Askar N., Itil I. (2015). Does pelvic floor muscle training abolish symptoms of urinary incontinence? A randomized controlled trial. Clin. Rehabil..

[25] Rahmani N., Mohseni-Bandpei M.A. (2011). Application of perineometer in the assessment of pelvic floor muscle strength and endurance: A reliability study. J. Bodyw. Mov. Ther..

[26] Alves F.K., Riccetto C., Adami D.B., Marques J., Pereira L.C., Palma P., Botelho S. (2015). A pelvic floor muscle training program in postmenopausal women: A randomized controlled trial. Maturitas.

[27] Avery K., Donovan J., Peters T.J., Shaw C., Gotoh M., Abrams P. (2004). ICIQ: A brief and robust measure for evaluating the symptoms and impact of urinary incontinence. Neurourol. Urodyn..

[28] Volløyhaug I., Mørkved S., Salvesen Ø., Salvesen K.Å. (2016). Assessment of pelvic floor muscle contraction with palpation, perineometry and transperineal ultrasound: A cross-sectional study. Ultrasound Obstet. Gynecol..

[29] Nyhus M.Ø., Oversand S.H., Salvesen Ø., Salvesen K.Å., Mathew S., Volløyhaug I. (2020). Ultrasound assessment of pelvic floor muscle contraction: Reliability and development of an ultrasound-based contraction scale. Ultrasound Obstet. Gynecol..

[30] Frawley H., Shelly B., Morin M., Bernard S., Bø K., Digesu G.A., Dickinson T., Goonewardene S., McClurg D., Rahnama’I M.S. (2021). An International Continence Society (ICS) report on the terminology for pelvic floor muscle assessment. Neurourol. Urodyn..

[31] Jagadeeswari J., KalaBarathi S. (2019). Effectiveness of vaginal cone therapy on urinary incontinence among women in saveetha medical college hospital, Thandalam, Chennai. Asian J. Pharm. Clin. Res..

[32] Bø K., Talseth T., Holme I. (1999). Single blind, randomised controlled trial of pelvic floor exercises, electrical stimulation, vaginal cones, and no treatment in management of genuine stress incontinence in women. BMJ.

[33] Pereira V.S., de Melo M.V., Correia G.N., Driusso P. (2012). Vaginal cone for postmenopausal women with stress urinary incontinence: Randomized, controlled trial. Climacteric.

[34] Seo J.T., Yoon H., Kim Y.H. (2004). A randomized prospective study comparing new vaginal cone and FES-Biofeedback. Yonsei Med. J..

[35] Arvonen T., Fianu-Jonasson A., Tyni-Lenné R. (2001). Effectiveness of two conservative modes of physical therapy in women with urinary stress incontinence. Neurourol. Urodyn..

[36] Hahn I., Milsom I., Ohlsson B.L., Ekelund P., Uhlemann C., Fall M. (1996). Comparative assessment of pelvic floor function using vaginal cones, vaginal digital palpation and vaginal pressure measurements. Gynecol. Obstet. Investig..

[37] Cervigni M., Gambacciani M. (2015). Female urinary stress incontinence. Climacteric.

[38] Farzinmehr A., Moezy A., Koohpayehzadeh J., Kashanian M. (2015). A Comparative study of whole body vibration training and pelvic floor muscle training on women’s stress urinary incontinence: Three- month follow-up. J. Family Reprod. Health.

[39] Franco P.G., Santos K.B., Rodacki A.L.F. (2015). Joint positioning sense, perceived force level and two-point discrimination tests of young and active elderly adults. Braz. J. Phys. Ther..

[40] Leech K.A., Roemmich R.T., Gordon J., Reisman D.S., Cherry-Allen K.M. (2022). Updates in motor learning: Implications for physical therapist practice and education. Phys. Ther..

[41] Cho S.T., Kim K.H. (2021). Pelvic floor muscle exercise and training for coping with urinary incontinence. J. Exerc. Rehabil..

[42] Rocca Rossetti S. (2016). Functional anatomy of pelvic floor. Arch. Ital. Urol. Androl..

